# Cannabinoid Use for Pain Reduction in Spinal Cord Injuries: A Meta-Analysis of Randomized Controlled Trials

**DOI:** 10.3389/fphar.2022.866235

**Published:** 2022-04-28

**Authors:** Sung Huang Laurent Tsai, Chun-Ru Lin, Shih-Chieh Shao, Chao-Hua Fang, Tsai-Sheng Fu, Tung-Yi Lin, Yu-Chiang Hung

**Affiliations:** ^1^ Department of Orthopaedic Surgery, Chang Gung Memorial Hospital, Chang Gung University, Keelung, Taiwan; ^2^ School of Medicine, College of Medicine, Fu Jen Catholic University, New Taipei, Taiwan; ^3^ Department of Pharmacy, Chang Gung Memorial Hospital, Keelung, Taiwan; ^4^ Department of Traditional Chinese Medicine, Chang Gung Memorial Hospital, Keelung, Taiwan; ^5^ Department of Chinese Medicine, Kaohsiung Chang Gung Memorial Hospital, Chang Gung University College of Medicine, Kaohsiung, Taiwan

**Keywords:** cannabinoids, spinal cord injury, trauma, spine, pain, adverse events

## Abstract

**Background:** Spinal cord injury (SCI) often involves multimodal pain control. This study aims to evaluate the efficacy and safety of cannabinoid use for the reduction of pain in SCI patients.

**Methods and Findings:** This study followed the Preferred Reporting Items for Systematic reviews and Meta-Analyses (PRISMA) statement. We searched PubMed, EMBASE, Scopus, Cochrane, Web of Science, and ClinicalTrials.gov for relevant randomized controlled trials (RCTs) reporting the efficacy (e.g., pain relief) or safety (e.g., adverse events) of cannabinoids in patients with SCI, from inception to 25 December 2021. The study quality and the quality of evidence were evaluated by Cochrane ROB 2.0 and the Grading of Recommendations, Assessment, Development, and Evaluations system (GRADE), respectively. We used the random-effects model to perform the meta-analysis. From a total of 9,500 records, we included five RCTs with 417 SCI patients in the systematic review and meta-analysis. We judged all five of the included RCTs as being at high risk of bias. This meta-analysis indicated no significant difference in pain relief between the cannabinoids and placebo in SCI patients (mean difference of mean differences of pain scores: −5.68; 95% CI: −13.09, 1.73; *p* = 0.13; quality of evidence: very low), but higher odds of adverse events were found in SCI patients receiving cannabinoids (odds ratio: 3.76; 95% CI: 1.98, 7.13; *p* < 0.0001; quality of evidence: moderate).

**Conclusion:** The current best evidence suggests that cannabinoids may not be beneficial for pain relief in SCI patients, but they do increase the risks of adverse events, including dizziness, somnolence, and dysgeusia, compared to the placebo. Cannabinoids should not be regularly suggested for pain reduction in SCI patients. Updating the systematic reviews and meta-analyses by integrating future RCTs is necessary to confirm these findings.

## Introduction

Pain represents the most common and debilitating consequence of spinal cord injuries (SCIs) and leads to poor quality of life (Burchiel and Hsu, 2001). Around the world, around 250,000 to 500,000 people suffer from SCI per year, and the annual global incidence is approximately 40–80 cases per million (Bickenbach et al., 2013). The lifetime costs per patient with SCI can reach $1.1–4.6 million (Ahuja et al., 2017). The treatment strategies for SCI patients may include control of blood pressure, use of corticosteroids, spinal immobilization, surgical intervention, anticoagulation prophylaxis, and sufficient pain management (Burchiel and Hsu, 2001; Wilson et al., 2013; Ahuja et al., 2017; Fehlings et al., 2017). Successful pain control in these patients could improve clinical outcomes, reduce hospital stay, lower the medical costs, and increase the quality of life (Burchiel and Hsu, 2001). Several multimodal analgesic agents, including opioids, gabapentinoids, *a*-adrenergic antagonists, antidepressants, and anticonvulsants, are widely used for pain management in SCI patients, but the overall effectiveness is suboptimal (Shiao and Lee-Kubli, 2018).

Cannabinoids, the active herbal compounds in cannabis, including tetrahydrocannabinol, dimethylheptylpyran, and parahexyl, have been present in Central Asia dating back 12,000 years (Andre et al., 2016; Crocq, 2020). Since then, the medicinal use of cannabis has been recorded in China, Egypt, Greece, and the Roman Empire. In China, cannabinoids were prescribed for anesthetic use from 221 B.C. to A.D. 220 (Crocq, 2020). In 1964, Raphael Mechoulam and Yechiel Gaoni identified THC in the cannabis sativa plant (Gaoni and Mechoulam, 1964). Subsequently, in 1967, Mechoulam R., Braun P. and Gaoni Y. synthesized THC (Mechoulam et al., 1967). Smoking or oral ingestion of cannabinoids may produce analgesic, antianxiety, antispasmodic, muscle relaxant, anti-inflammatory, and anticonvulsant effects (Andre et al., 2016). This medication may help SCI patients with pain reduction since cannabinoids may cause an antinociceptive effect by activating TRPA1, TRPV1, TRPV2, TRPV4, and G-protein–coupled receptors (Hill, 2015; Mlost et al., 2020). Currently, scientists are investigating the potential use of cannabinoids for pain reduction. However, cannabinoids are not risk-free. For example, in animal studies, the adverse effects or toxicity included neurotoxicity, hepatocellular injuries, developmental toxicity, embryo–fetal mortality, spermatogenesis reduction, organ weight alterations, male reproductive system alterations, and hypotension (Huestis et al., 2019). Another previous review study also reported the adverse effects of cannabinoids, including diarrhea, hepatic abnormalities, fatigue, vomiting, and somnolence, in humans (Huestis et al., 2019)**.** Some studies have demonstrated that cannabinoids are effective for chronic pain, neuropathic pain, and spasticity, but the efficacy and safety of cannabinoids use in SCI patients have not been systematically evaluated. Therefore, in this study, we surveyed the existing literature to estimate the degree of pain relief and adverse events derived from cannabinoid use in SCI patients.

## Methods

### Research Protocol and Search Question

We conducted this study following the Preferred Reporting Items for Systematic reviews and Meta-Analyses (PRISMA) statement guidelines. The study protocol of this systematic review and meta-analysis has been registered in PROSPERO (CRD42022304188). We narrowed our study question by patients, interventions, comparisons, and outcomes to include patients with SCI, the use of cannabinoids versus placebo, and pain controls or adverse events.

### Eligibility Criteria and Primary Outcome

We included the studies based on the following inclusion criteria: 1) Patients with SCIs as the study population, 2) only randomized controlled study as the design, and 3) pain scores (Visual Analog Scale, VAS or Numerical Rating Scale, NRS) or adverse events as the study outcomes. We excluded studies that were 1) single-arm follow-up studies; (2), case reports, case series, reviews, basic science experiments, or nonhuman studies; and 3) conference abstracts.

### Search Strategy and Study Selection

On 25 December 2021, we searched PubMed, Embase, Scopus, The Cochrane Library, Web of Science, and ClinicalTrials.gov for articles by using the combination of keyword and medical subject heading (MeSH) or Emtree terms for each database. To make our search more comprehensive, we also searched the reference lists in the included studies. Two independent reviewers (CRL and SHLT) screened the titles and abstracts for possible eligibility and then independently read the full-text articles to determine the eligibility for final inclusion. All disagreements between the reviewers were resolved through discussion.

### Data Collection and Quality Assessment

The following data were extracted by two reviewers (CRL and SHLT): study characteristics (author, year of publication, region of study, data source, study design, and period of study), study arms, sample size, patient age, inclusion criteria of each study, the specific definition of each treatment arm, and the outcomes of interest including pain scales and adverse events. The two reviewers (CRL and SHLT) independently assessed the risk of bias in the included studies and quality of evidence in the study outcomes by using Cochrane ROB 2.0 and the GRADE (Grading of Recommendations, Assessment, Development, and Evaluations) system (Goldet and Howick, 2013; Higgins et al., 2019a; Higgins et al., 2019b). All discrepancies were resolved by discussion.

### Statistical Analysis and Quantitative Data Synthesis

We performed a pairwise meta-analysis to compare the efficacy and safety between cannabinoids and placebo in SCI patients. With regard to pain control, mean differences (MDs) were used to calculate the mean differences in treatment responses attributed to cannabinoids. With regard to adverse events, odd ratios were used to calculate the risk of adverse events attributed to cannabinoids. To measure statistical heterogeneity for the result estimates, we defined I^2^ of 25–50%, 51–75%, and 76–100% as low, moderate, and high statistical heterogeneity (DerSimonian and Laird, 1986), respectively. Since we anticipated clinical heterogeneity between the included studies, we used the random-effects model to estimate the pooled results in the meta-analysis. A *p*-value <0.05 was considered statistically significant in all the analyses.

## Results

### Literature Search and Selection Process

**FIGURE 1:**
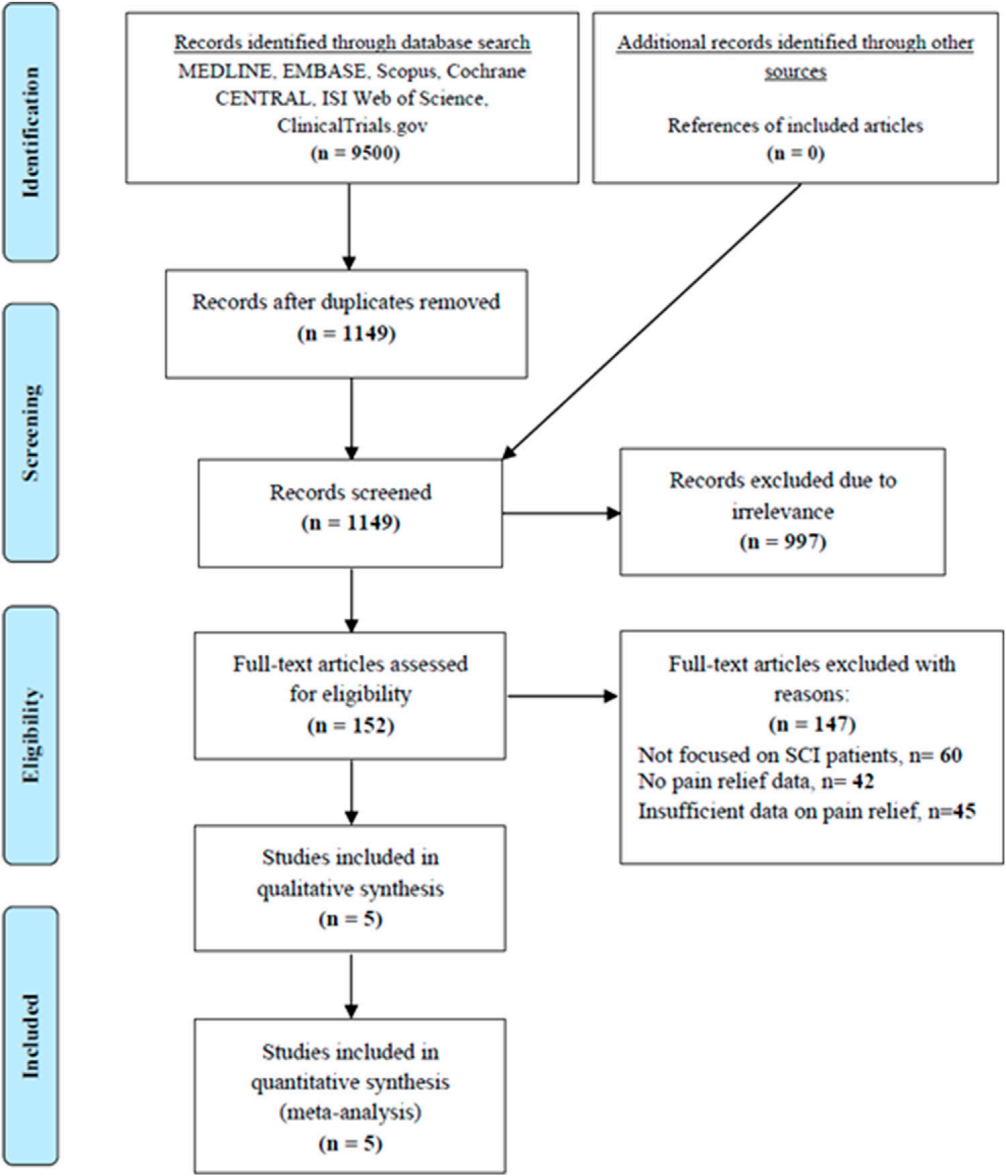
Flow diagram of identification, screening, eligibility, and inclusion.

We initially found a total of 9,500 records through different electronic database searches. After removing duplicate and irrelevant studies by screening for titles and abstracts, we identified 152 full-text articles eligible for inclusion, of which five RCTs with 417 participants were ultimately included in this meta-analysis (Figure 1).

### Study Characteristics

**FIGURE 2:**
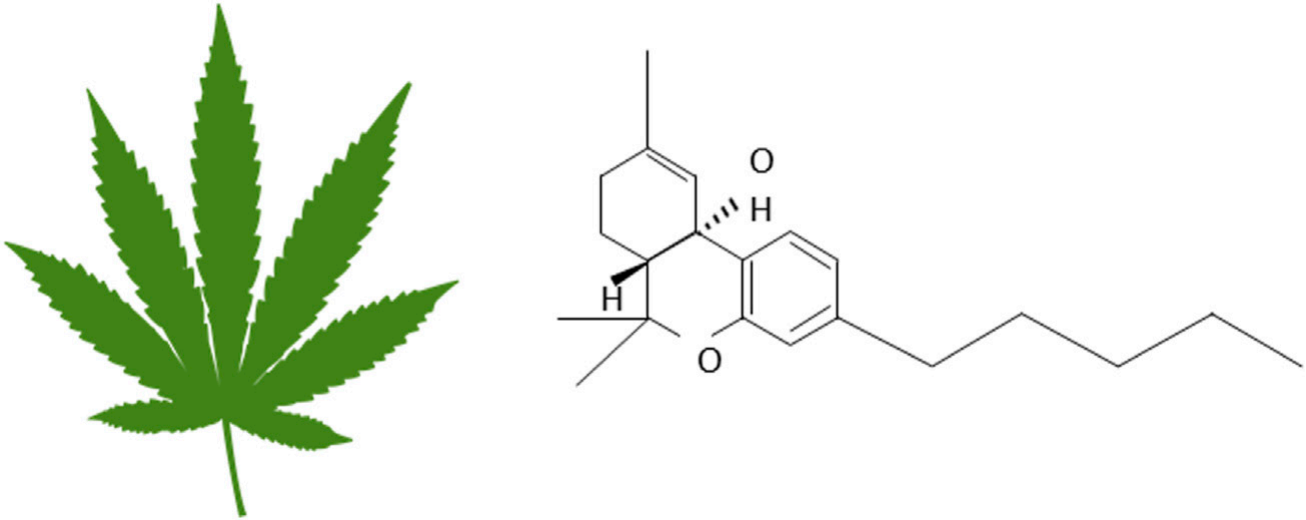
Illustration of cannabinoids and the chemical formula of the main ingredient THC.

Table 1 summarizes the study characteristics of the included studies. These studies were from the United Kingdom, the United States, Israel, and Denmark (Berman et al., 2004; Wilsey et al., 2008; Andresen et al., 2016; NCT, 2018; Weizman et al., 2018). Four studies were published as full articles (Berman et al., 2004; Wilsey et al., 2008; Andresen et al., 2016; Weizman et al., 2018), while one study was registered on Clinicaltrials.gov (NCT, 2018)**.** One study included male participants only (Andresen et al., 2016). One, two, and two studies, respectively, used inhaled cannabinoids (Wilsey et al., 2008), oromucosal sprayed cannabinoids (Berman et al., 2004; NCT, 2018), and sublingual cannabinoids (Andresen et al., 2016; Weizman et al., 2018) as the treatment. Two studies contained two intervention groups, including that by Wilsey et al. (Wilsey et al., 2008) (high and low dose THC) and Berman et al. (Berman et al., 2004) (THC and nabiximols). The characteristics of the formulations, compounds, and concentrations in the included studies are summarized in Tables 2, 3. Figure 2 depicts the cannabis leaf and chemical structure of THC.

**TABLE 1:** Study characteristics of the included studies.

Study	Design	Location	Drug type	Inclusion criteria	Experimental group 1	Experimental group 2	Control group	Age (yrs)	Sex (M/F)	Outcome
Berman et al. (2004)	RCT	United Kingdom	Oromucosal	Brachial plexus root avulsion	Nabiximols, 46 participants	THC 27 mg/ml, 47 participants	Placebo, 48 participants	23–69	46/2	Pain severity (NRS, BS-11), sleep quality (BS-11), sleep disturbance, pain-related quality of life (SF-MPQ, PDI, GHQ-12)
					1. Eight sprays at any one time or within a 3 h period. 2. 48 sprays within any 24 h period	1. Eight sprays at any one time or within a 3 h period. 2. 48 sprays within any 24 h period				
Wilsey et al. (2008)	RCT	United States	Inhalation	CRPS type I, SCI, peripheral neuropathy, or nerve injury	7% cannabis 490 mg, 33 participants	3.5% cannabis 550 mg, 36 participants	Placebo, 34 participants	46 ± 25	20/18	Pain intensity (VAS), pain unpleasantness (VAS), PGIC, NPS, allodynia (VAS), heatpain threshold (Medoc TSA 2001 Peltier thermode), psychoactive effects, mood (VAS), neurocognitive assessments (WAIS-III, pen and paper test, HVLT, GPT, A–F), neuropsychological tests (HVLT)
NCT01606202 2018	RCT	United Kingdom	Oromucosal	CNP in SCI	Nabiximols, 49 participants	N/A	Placebo, 57 participants	48.1 ± 12.69	91/25	Pain intensity (NRS), use of rescue analgesia, spasm severity (NRS), days on spasm, MAS, SOMC, Spitzer-QLI, CSI, PGIC, BPI-SF, sleep disturbance (NRS)
					1.Maximum permitted dose was 48 actuations in 24 hrs					
Andresen et al. (2016)	RCT	Denmark	Sublingual	SCI including caudal equine lesions, with NP	PEA-um, 36 participants	N/A	Placebo, 37 participants	56.3 ± 11.6	54/19	Pain intensity (NRS, NPS), spasticity (NRS), sleep disturbance (NRS), the intensity of muscle stiffness and spasms, health-related quality of life, PGIC
					1.600 mg*BID					
Weizman et al. (2018)	RCT	Israel	Sublingually	Chronic lumbar radicular pain	THC oil, 15 participants	N/A	Placebo, 15 participants	33.3 ± 3.9	17/0	Visual analog scale (VAS) score, fMRI
					1. Average THC dosage = 15.4 ± 2.2 mg					

**TABLE 2:** Patented formulations, botanical or chemical.

Study	Formulation	Source	Species, concentration	Quality control reported? (Y/N)	Chemical analysis reported? (Y/N)
Berman et al. (2004)	27 mg/ml delta-9-tetrahydrocannabinol and 25 mg/ml cannabidiol	GW Pharma Ltd.	38–44 mg and 35–42 mg of two extracts (as soft extracts) from *Cannabis sativa* L., folium cum flore (cannabis leaf and flower)	Y	Y
NCT01606202 (2018)	27 mg/ml delta-9-tetrahydrocannabinol and 25 mg/ml cannabidiol	GW Pharma Ltd.	38–44 mg and 35–42 mg of two extracts (as soft extracts) from *Cannabis sativa* L., folium cum flore (cannabis leaf and flower)	Y	Y

**TABLE 3:** Isolated chemical compound.

Study	Compound, concentration	Source	Purity (%) (and grade, if applicable)	Quality control reported? (Y/N)
Berman et al. (2004)	delta-9-tetrahydrocannabinol, 27 mg/ml	Purified by Berman et al. (2004)	(≥90%)	N
Wilsey et al. (2008)	delta-9-tetrahydrocannabinol, 3.5 and 7%	Purified by the University of Mississippi (2008)	(≥90%)	Y
Andresen et al. (2016)	Ultramicronized palmitoylethanolamide, 600 mg	Epitech Group SpA	(≥90%)	Y
Weizman et al. (2018)	delta-9-tetrahydrocannabinol oil, 0.2 mg/kg	Panaxia Pharmaceutical Industries, Lod	(≥90%)	Y

### Methodological Quality and Assessment of Risk of Bias

**FIGURE 3:**
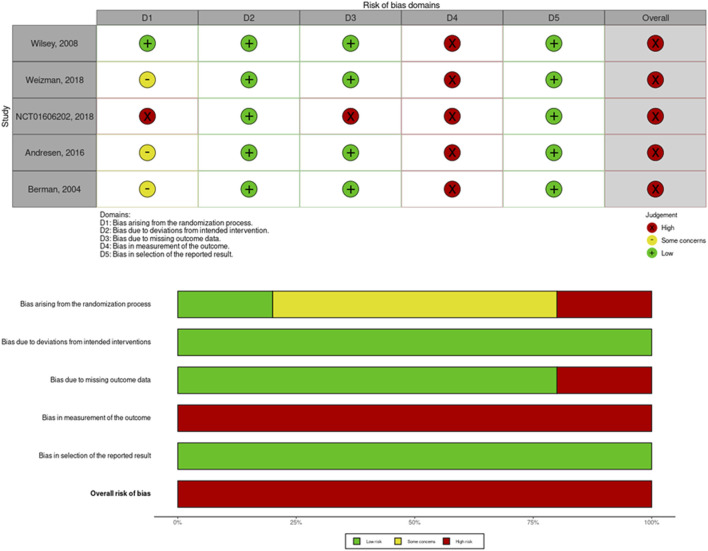
ROB2, assessment of risk of bias in the included studies and the summary of domains.

Based on ROB 2.0, five RCTs were rated with an overall high risk of bias **(** Figure 3). Regarding the risk of bias arising from the randomization process, three studies did not exhibit a baseline balance of the demographic characteristics (Berman et al., 2004; Andresen et al., 2016; Weizman et al., 2018), which was rated as raising some concerns. Furthermore, one study failed to report the baseline NRS data of its placebo and cannabinoid groups, and therefore, a high risk of bias may have been introduced through the randomization process (NCT, 2018). Regarding the risk of bias from the missing outcome data, one study failed to explain the reasons and did not report the numbers lost to follow-up and was hence rated as being at high risk of bias (NCT, 2018). Regarding the measurement of outcome data, five RCTs used the VAS or NRS score. Both are subjective patient-reported outcome data to assess the degree of pain and may carry a high risk of bias (Berman et al., 2004; Wilsey et al., 2008; Andresen et al., 2016; NCT, 2018; Weizman et al., 2018). The GRADE assessment is summarized in Table 4.

**TABLE 4:** GRADE (Grading of Recommendations, Assessment, Development and Evaluations) criteria for assessing the quality of evidence.

Outcome	Number of studies	Number of participants	Risk of bias	Imprecision	Inconsistency	Indirectness	Publication bias	Relative effect (95% confidence interval)	Confidence in effect estimate (Grade)
Analgesic effect	4	276	Serious	Serious	Serious	Not serious	Not serious	−5.68 (−13.09, 1.73)	Very low
Adverse effect	3	368	Serious	Serious	Not serious	Not serious	Not serious	3.85 (2.11, 7.18)	Moderate

### Pain

**FIGURE 4:**
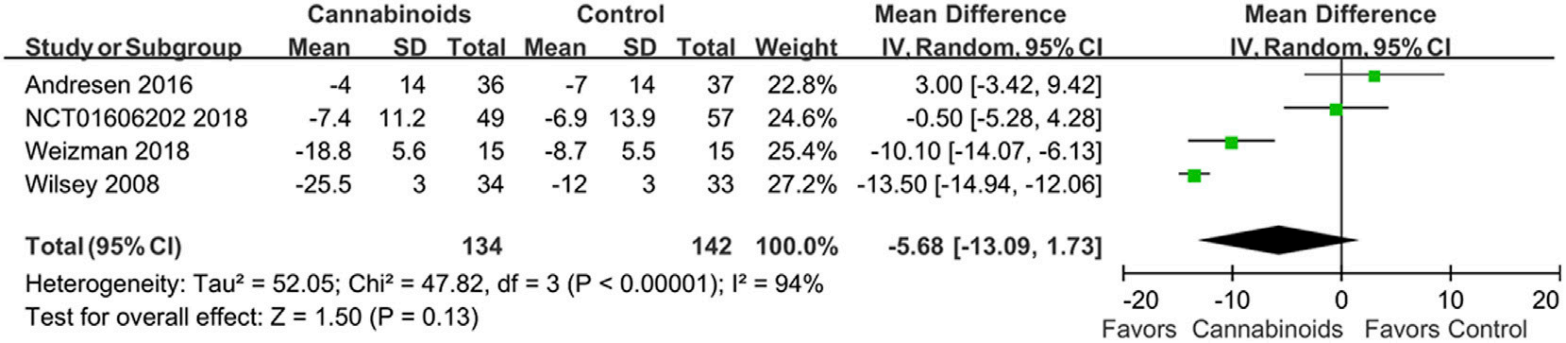
Forest plot showing overall pain scores when comparing cannabinoids and placebo. Better pain control is shown by the favored side of the plot.

Four RCTs with 276 SCI patients were included to assess the treatment efficacy of cannabinoids (Wilsey et al., 2008; Andresen et al., 2016; NCT, 2018; Weizman et al., 2018) In Figure 4, we show conflicting results regarding pain reduction in SCI patients using cannabinoids or placebo. For example, the study by Andresen et al. indicated that cannabinoids had no significant differences in pain reduction compared to placebo (Andresen et al., 2016), but Weizman et al. and Wilsey et al. indicated that cannabinoids reduced pain compared to placebo (Weizman et al., 2018). However, our meta-analysis did not find a statistically significant difference in pain control for SCI patients between cannabinoids and placebo (MD of MDs -5.68; 95% CI: −13.09, 1.73, *p* = 0.13; I^2^: 94%; quality of evidence: very low) (Figure 4).

### Adverse Effects

**FIGURE 5:**
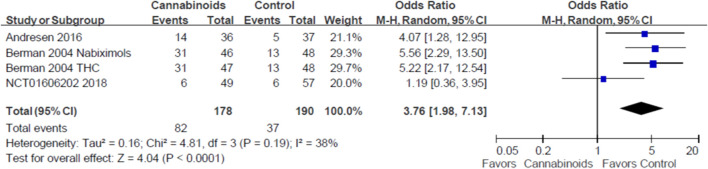
Forest plot showing overall adverse events when comparing cannabinoids and placebo. Lower rate of adverse events is shown by the favored side of the plot.

Three studies with 320 SCI patients reported the treatment safety of cannabinoids (Berman et al., 2004; Andresen et al., 2016; NCT, 2018). In Figure 5, we show conflicting results regarding the risk of any adverse events in SCI patients using cannabinoids or placebo. For example, the studies by Andresen et al. and Berman et al. indicated that cannabinoids increased the risk of any adverse events (Berman et al., 2004; Andresen et al., 2016), but NCT01606202 indicated that cannabinoids did not affect the risk of any adverse events (NCT, 2018). However, our meta-analysis found a statistically significant risk of any adverse events for SCI patients using cannabinoids, compared to placebo (odds ratio, OR: 3.76; 95% CI: 1.98, 7.13; p < 0.0001, quality of evidence: moderate). We summarize the reported adverse events in Table 5.

**TABLE 5:** Adverse events comparing cannabinoids and placebo.

Any adverse events	Cannabinoid group (n = 178)	Placebo group (n = 143)
Nervous system	49(27.5%)	12(8.5%)
Dizziness	20(11.2%))	5(3.5%)
Somnolence	13(7.3%)	5(3.5%)
Dysgeusia	15(8.4%)	1(0.7%)
Confusion	1(0.5%)	0(0.0%)
Blurred vision	0(0.S0%)	1(0.7%)
Gastrointestinal	12(6.7%)	5(3.5%)
Nausea	6(3.3%)	3(2.1%)
Paralytic ileus	3(1.7%)	1(0.7%)
Cholecystolithiasis	3(1.7%)	1(0.7%)
Psychiatry/mood	9(5.0%)	0(0.0%)
Feeling drunk	8(4.4%)	0(0.0%)
Paranoia	1(0.6%)	0(0.0%)
Immune system/infection	8(4.4%)	4(2.8%)
Methicillin-resistant Staphylococcus aureus infection	1(0.6%)	0(0.0%)
Urinary tract infection	3(1.7%)	2(1.4%)
Pneumonia	0(0%)	1(0.7%)
Erysipelas	3(1.7%)	1(0.7%)
Fungus infection	1(0.6%)	0(0.0%)
Osteopathy	1(0.6%)	1(0.7%)
Tibia Fracture	1(0.6%)	0(0.0%)
Upper Limb Fracture	0(0%)	1(0.7%)
General disorder	3(1.7%)	1(0.7%)
Fall	1(0.6%)	1(0.7%)
Anaemia	1(0.6%)	0(0.0%)
suicide	1(0.6%)	0(0.0%)
Skin and subcutaneous tissue disorders	0(0.0%)	1(0.7%)
Contusion	0(0.0%)	1(0.7%)

### *Post Hoc* Analysis

**FIGURE 6:**
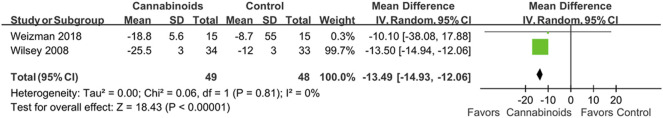
Post hoc analysis comparing cannabinoids to placebo after including only studies reporting VAS as pain scores. Better pain control is shown by the favored side of the plot.

**FIGURE 7:**
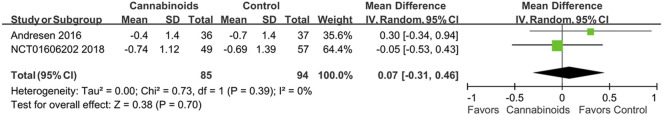
Post hoc analysis comparing cannabinoids to placebo after including only studies reporting NRS as pain scores. Better pain control is shown by the favored side of the plot.

**FIGURE 8:**
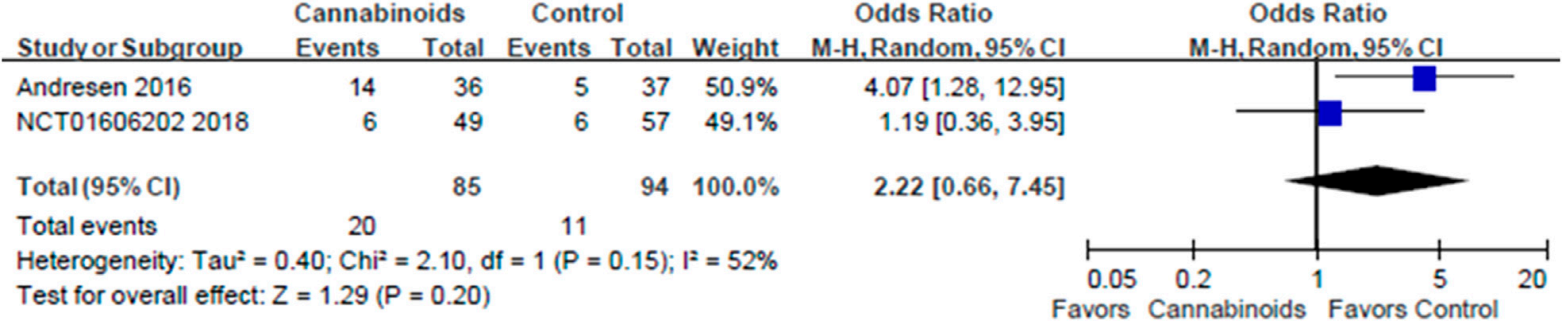
Post hoc analysis comparing cannabinoids to placebo after excluding the study population with brachial plexus avulsion among the adverse events. Lower rate of adverse events is shown by the favored side of the plot.

The first *post hoc* analysis included two outcome subgroups, the VAS score and NRS score groups, for analgesic effects. Two studies used VAS scores to evaluate the analgesic effects of cannabinoids (Wilsey et al., 2008; Weizman et al., 2018). As shown in Figure 6, we did find a statistically significant difference in pain control for SCI patients between cannabinoids and placebo in the studies with the VAS outcome (MD of MDs: 13.49, 95% CI: −14.93, −12.06, *p* < 0.00001; I^2^; 0%; quality of evidence: very low). Two studies used the NRS scores to evaluate the analgesic effects of cannabinoids (Andresen et al., 2016; NCT, 2018). As shown in Figure 7, we did not find a statistically significant difference in pain control for SCI patients between cannabinoids and placebo in the studies with the NRS outcome (MD of MDs: 0.07, 95% CI: −0.31, 0.46; *p* = 0.70; quality of evidence: very low). Another post hoc analysis for adverse effects included two studies (Andresen et al., 2016; NCT, 2018) but excluded one study in which the patients had brachial plexus root avulsion (Weizman et al., 2018). As can be seen in Figure 8, we did not find a statistically significant difference in the adverse effects for SCI patients between cannabinoids and placebo (OR 2.22, 95% CI 0.66, 7.45, p = 0.20, quality of evidence: moderate).

## Discussion

Our study did not demonstrate a better analgesic effect of cannabinoids than that of placebo in SCI patients. In fact, the adverse events were higher in the cannabinoid group than in the placebo group. The majority of these side effects involved the nervous system, such as dizziness, somnolence, and dysgeusia. To the best of our knowledge, this may be the first systematic review and meta-analysis to compare the analgesic effects and adverse events of cannabinoids in SCI patients.

The SCI-induced pain includes nociceptive (musculoskeletal or visceral), neuropathic (at level or below level), and other types of pain (Bryce et al., 2012). According to the International Association for the Study of Pain, neuropathic pain is defined as “pain initiated or caused by a primary lesion, dysfunction, or transitory perturbation of the peripheral or central nervous system” (Classification of chronic pain, 1986). Neuropathic pain can be classified into peripheral and central types. Central neuropathic pain occurs after spinal cord injury, stroke, and multiple sclerosis. Peripheral neuropathic pain commonly occurs in conjunction with diseases resulting in peripheral nerve damage such as cancer and diabetes. SCI causes both central and peripheral neuropathic pain and is usually challenging for clinicians to manage (Canavero and Bonicalzi, 2011). Canavero and Boncalzi reviewed the efficacy of cannabinoids and other medications for central neuropathic pain (Canavero and Bonicalzi, 2018). They concluded that cannabinoids were not better than the other commonly used drugs and do not support a role in the management of central pain. The common medications including opioids, gabapentinoids, and NSAIDS were also not beneficial for the treatment of central pain. Even for multiple sclerosis, which is considered a subtype of cord central pain, the in-depth review by Canavero and Bonicalzi did not find evidence for a major effect (Canavero and Bonicalzi, 2018).

Cannabinoids can interact with the CB_1_ receptors, CB_2_ receptors, *N*-arachidonoyl glycine (NAGly) receptors, and opioid or serotonin (5-HT) receptors, which produce analgesic effects (Vučković et al., 2018). CB_1_ receptors are commonly found in the brain, spinal cord, and peripheral nervous systems (Pertwee, 1997). CB_2_ receptors are found in immune cells, and cannabinoids may induce anti-inflammatory and analgesic effects while acting on human immune cells (Galiègue et al., 1995). Previous studies reported that both CB_1_ and CB_2_ receptors could upregulate in the nervous structures when the nerve is damaged, explaining the advantages of cannabinoids for pain relief (Lim et al., 2003; Arevalo-Martin et al., 2016). This may be helpful in treating SCI pain, as the upregulated system may counteract the damage of the nerve structure (Arevalo-Martin et al., 2016). Cannabinoids also interact with various neurotransmitters and neuromodulators including acetylcholine, dopamine, γ-aminobutyric acid (GABA), histamine, serotonin, glutamate, norepinephrine, prostaglandins, and opioid peptides. Pharmacologic effects on movement and spastic disorders, which may present in SCI patients, are activated by the interactions with the GABAergic, glutaminergic, and dopaminergic transmitter systems (Musty and Consroe, 2002). However, a pilot study of only five participants reported that cannabinoids did not have a significant analgesic effect compared to that of placebo (Rintala et al., 2010). Similarly, our study showed that compared to placebo, the analgesic effects of the cannabinoids were not beneficial in treating SCI pain. However, this may have resulted from a lack of high-quality evidence. Only four studies were allowed for the meta-analysis. Wilsey et el. reported significant pain reduction in cannabinoids compared to placebo. However, the study was published in 2008 (Wilsey et el., 2008). In fact, the only study that concluded no statistically significant adverse event compared to placebo was the study NCT01606202 2018, which was only registered on Clinicaltrials.gov and never published, even after years (NCT, 2018).

The most common adverse events of cannabinoids involved the nervous system, constituting 27.5% (n = 49) in the cannabinoid group. Among these, the most common adverse events were dizziness (11.2%, *n* = 20), somnolence (7.3%, *n* = 13), and dysgeusia (8.4%, n = 15). and confusion (0.5%, n = 1) in the cannabinoid group. All the adverse events had higher prevalence in the cannabinoid group. Somnolence could be explained by the sleep-inducing effect of cannabinoids. Cannabinoids may decrease sleep onset latency, decrease waking after sleep onset, increase slow-wave sleep, and decrease REM sleep (Pivik et al., 1972; Feinberg et al., 1976). These effects are caused by the cannabinoids signaling on CB_1_ receptors. Similarly, Huestis et al. reported that cannabinoids may cause severe adverse events, including somnolence, fatigue, diarrhea, vomiting, hepatic abnormalities, central nervous system inhibition, neurotoxicity, and hypotension (Huestis et al., 2019).

A previous meta-analysis from Aviram and Samuelly-Leichtag in 2017 explored the analgesic effect of cannabinoids in chronic pain. They reported that cannabinoids could relieve chronic pain, especially neuropathic pain (Aviram and Samuelly-Leichtag, 2017). However, the study did not focus on SCI-related neuropathic pain and pooled all chronic neuropathic pain in their analyses. A 2019 meta-analysis explored the relationship between the analgesic effects of different cannabinoids and neuropathic pain. However, they also did not specify the cause of the neuropathic pain in their analyses (Rabgay et al., 2020). Our meta-analysis focused on the analgesic effects and adverse effects of cannabinoids in SCI pain, and may therefore guide clinicians in the management of SCI.

There are few meta-analyses exploring the relationship between cannabinoids and neuropathic pain, which are not focused on SCI. The systematic search strategy to identify high-quality research allowed us to make an assessment of the study quality. However, the clinical heterogeneity among the included studies should be noted when interpreting our study findings. For example, some studies included SCI patients with peripheral neuropathic pain, while others included patients with central neuropathic pain. In addition, the included studies used various forms of cannabinoid drugs and treatment dosages, which may affect the certainty of our pooled results. However, in order to address this issue, we performed the meta-analysis using a random-effects model and applied the GRADE system to judge the certainty of the evidence.

## Conclusion

Our systematic review and meta-analysis of RCTs suggested that cannabinoids, compared to placebo, have no clinically significant benefits for pain reduction among SCI patients but may have higher rates of adverse events, including dizziness, somnolence, and dysgeusia. Considering that the certainty of the evidence remains suboptimal due to the risk of bias, small sample sizes, and inconsistencies among the included studies, more RCTs are necessary to confirm our findings.

## Data Availability

The original contributions presented in the study are included in the article/Supplementary Material, further inquiries can be directed to the corresponding authors.
